# Genome report of a probiotic *Bacillus subtilis* strain isolated from healthy human feces in Vietnam

**DOI:** 10.1128/mra.00513-25

**Published:** 2025-08-25

**Authors:** Hoi Le Thi, Hoa Hoang Thi Thanh, Bach Dao Gia, Linh Doan Thi Thuy, Ngan Luu Thi Thuy, An Nguyen Huu, Le Bui Nhat, Tien Vuong Quang, Mai Dao Tuyet, Ba Binh Ngo, Trung Nguyen Vu

**Affiliations:** 1 Hanoi Medical University 106156, Hanoi, Vietnam; 2 Eramic Technology Era Joint Stock Company, Hanoi, Vietnam; 3 Hanoi University of Science and Technology 118018, Hanoi, Vietnam; 4 Faculty of Applied Sciences, International School, Vietnam National University 54799, Hanoi, Vietnam; 5 VNU University of Science 308298, Hanoi, Vietnam; 6 Pasteur Institute In Ho Chi Minh City 581174, Ho Chi Minh City, Vietnam; University of Maryland School of Medicine, Baltimore, Maryland, USA

**Keywords:** genome assembly, *Bacillus subtilis*, human feces, Vietnam

## Abstract

This report presents the genome sequence of *Bacillus subtilis* strain Eramic25, isolated from the fecal sample of a healthy adult in Vietnam. Genome analysis confirms its safety and reveals genes associated with probiotic characteristics, supporting its potential application in functional foods and human health.

## ANNOUNCEMENT

*Bacillus subtilis* is a gram-positive, spore-forming bacterium that transiently colonizes the human gut and is considered a probiotic due to its roles in digestion, immunity, and pathogen inhibition (1–3). It also influences the gut–brain axis, enhancing cognition and reducing anxiety (4–6). Industrially, it is used to produce enzymes and fermented foods (7–9). However, some strains harbor virulence genes. This study analyzes the draft genome of *B. subtilis* Eramic25 to evaluate its safety as a probiotic candidate (10, 11).

A stool sample was collected in 2022 from a healthy volunteer in Hanoi, Vietnam, under ethics approval code 637/QĐ-ĐHYHN. The sample was homogenized in sterile 1× PBS, heat-treated at 80°C for 30 min, diluted to 10⁻³, and plated on LB agar. After 24 h of incubation at 37°C, colonies with off-white, opaque, serrated morphology were isolated and identified as *B. subtilis* strain Eramic25 using MALDI-TOF MS. Genomic DNA was extracted using the PCG MagBead DNA Extraction Kit (Vietnam). A DNA library was prepared with the Nextera Kit, and sequencing was done on the Illumina MiSeq platform (2 × 75 bp) with size selection via AMPure XP Beads, generating 5,403,583 paired-end reads (~10.8 million total) and ~100× coverage. Quality control was performed using FastQC v0.12.1 (12) and Fastp v0.24.1 (13). Host reads were removed by mapping to the GRCh38 genome using Bowtie2 v1.3.1 (14–16).

**Fig 1:**
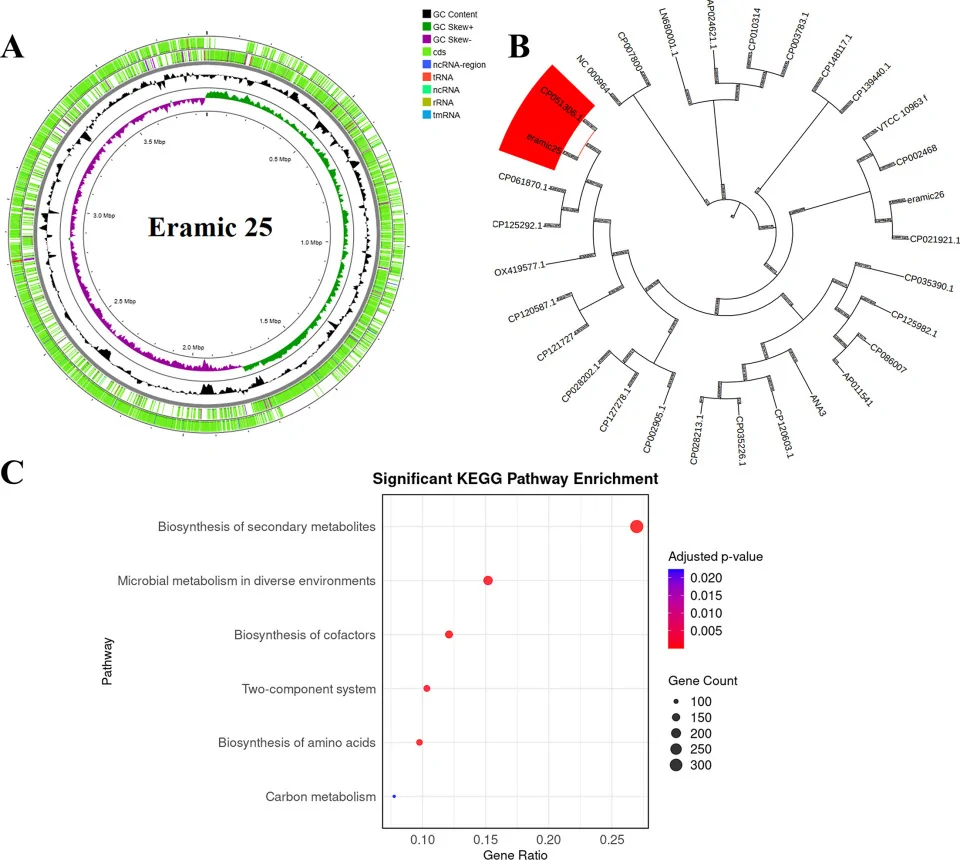
(**A**) Genome annotation using the RAST server; (**B**) Phylogenetic tree; (**C**) Functional enrichment analysis based on KEGG pathways of *B. subtilis* strain Eramic25.

The genome of *B. subtilis* strain Eramic25 was sequenced on the Illumina MiSeq platform and deposited in GenBank under accession number GCA_031932165.1 and project JAUTIS01. Default parameters were used for all software. After quality control, filtered reads were assembled *de novo* using SPAdes v3.15.5 (17), yielding a 3,855,385 bp draft genome with a GC content of 43.53%, 100 contigs, an N50 of 95,906 bp, and estimated coverage of 100×. Assembly quality and completeness (97.2%) were assessed using QUAST v5.0.2 (18). Genome annotation was performed using the RAST server, predicting 4,247 protein-coding sequences and 39 RNA genes (4 rRNA, 33 tRNA, and 1 tmRNA). Multilocus sequence typing (MLST) using MLST v2.23.0 (19) and the PubMLST database (20) identified the strain as sequence type ST123, with 100% identity across seven housekeeping genes: *glpF*(3), *ilvD*(31), *pta*(35), *purH*(40), *pycA*(56), *rpoD*(3), and *tpiA*(4). To investigate taxonomic placement, 30 *B. subtilis* strains (Table 1) were used for pangenome analysis with Roary v3.13.0 (21). Core genes were aligned with MAFFT v7.526 (22), and a maximum-likelihood phylogenetic tree was constructed using FastTree v2.1.11 (23) and visualized in iTOL (24). The tree revealed that Eramic25 is closely related to *B. subtilis* strain RI491 (CP051306.1), a known biofilm-forming strain. Contigs were reordered with RagTag (25) using *B. subtilis* RI491 as the reference genome. Functional annotation was conducted using eggNOG-mapper (26), and KEGG enrichment analysis revealed significant representation in pathways such as secondary metabolite biosynthesis, cofactor biosynthesis, amino acid metabolism, and two-component systems. Notably, no virulence-associated pathways were enriched (Fig. 1). Screening for virulence genes with ABRicate v1.0.1 (27) confirmed the absence of major toxin genes, including *bceT*, *hblABC*, *nheABC*, *cytK*, *entFM*, and *cesB*, supporting the strain’s probiotic safety profile.

**TABLE 1:** Selected *B. subtilis* strains for phylogenetic tree analysis

Genome (Accession number)	Genome size (Mb)	G + C content (%)	Genome (Accession number)	Genome size (Mb)	G + C content (%)
*B. subtilis* strain ANA3 (GCF_038397795.1*)*	3.99	44	*B. subtilis* subsp. *natto* strain SCP010-1 (GCA_020773035.1)	4.1	43.5
*B. subtilis* subsp. *natto* BEST195 (GCA_000209795.2)	4.1	43.5	*B. subtilis* strain PRO121 (GCA_029536835.2)	4.4	43
*B. subtilis* BEST3095 (GCA_019704335.1)	4.2	43.5	*B. subtilis* strain DSM 5611 (GCA_029536975.2)	4.2	43.5
*B. subtilis* BSn5 (GCA_000186745.1)	4.1	44	*B. subtilis* strain PRO61 (GCA_029762155.1)	4.0	43.5
*B. subtilis* subsp. *spizizenii* TU-B-10 (GCA_000227465.1)	4.2	44	*B. subtilis* strain SRCM117797 (GCA_030028395.1)	4.3	43.5
*B. subtilis* QB928 (GCA_000293765.1)	4.1	43.5	*B. subtilis* strain ZKY05 (GCA_046529455.1)	4.0	43.5
*B. subtilis* subsp. *subtilis* str. JH642 substr. AG174 (GCA_000699465.1)	4.1	43.5	*B. subtilis* strain KFRI-FA111 (GCA_030285965.1)	4.3	43.5
*B. subtilis* subsp. *subtilis* strain 3NA (GCA_000827065.1)	4.2	43.5	*B. subtilis* strain GUCC4 (GCA_034258595.1)	4.2	43.5
*B. subtilis* subsp*. subtilis* strain SRCM101392 (GCA_002202035.1)	4.1	44	Mutant *B. subtilis* isolate FELIX_MS509 (GCA_037751545.1)	4.2	43.5
*B. subtilis* strain SRCM102754 (GCA_009913275.1)	4.3	43.5	*B. subtilis* strain Eramic26 (GCA_031932205.1)	4.0	44
*B. subtilis* strain SRCM102749 (GCA_009913535.1)	4.2	43.5	*B. subtilis* strain BS34A (GCA_000952895.1)	4.2	43.5
*B. subtilis* strain SRCM103517 (GCA_004103535.1)	4.2	43.5	*B. subtilis* subsp. *subtilis* str. 168 (GCA_000009045.1)	4.2	43.5
*B. subtilis* strain SRCM103641 (GCA_004119555.1)	4.1	43.5	*B. subtilis* isolate NRS6120 (GCA_905312035.2)	4.2	43.5
*B. subtilis* strain RI4914 (GCA_012534935.1)	4.1	43.5	*B. subtilis* strain VTCC 10963 (GCA_036231745.1)	4.1	43.5
*B. subtilis* strain FX-1 (GCA_022647025.1)	4.3	43			

## Data Availability

The nucleotide sequence of *B. subtilis* Eramic 25 has been deposited in DDBJ/ENA/GenBank under accession number GCF_031932165.1. The raw reads are available under SRR Run accession number SRR33555242. The annotation of *B. subtilis* Eramic 25 by RAST is also available on figshare at B. subtilis_Eramic25-RAST_Annotation.
